# Supplementary material for: TDP-43 Proteinopathy Causes Broad Metabolic Alterations including TCA Cycle Intermediates and Dopamine Levels in Drosophila Models of ALS
Source: Metabolites. 2022 Jan 21;12(2):101. doi: 10.3390/metabo12020101 (PMC8876928; doi:10.3390/metabo12020101)
Supplement: Supplementary file 1 [file metabolites-12-00101-s001.zip › Supplemental Figure S1 and legend.pdf]

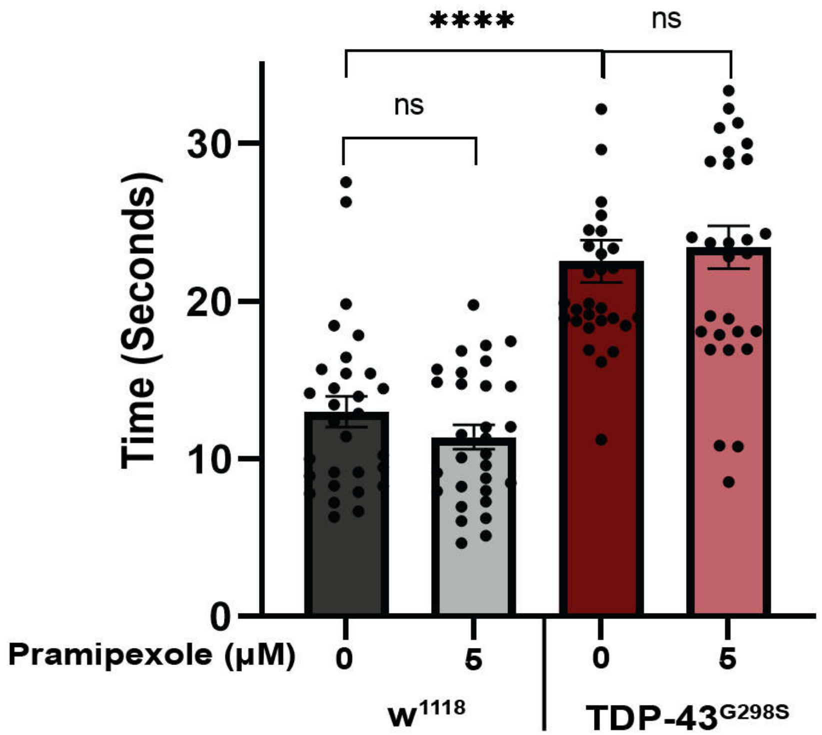

Figure S1: Larval turning assays for *Drosophila* expressing TDP-43^{G298S} fed a cornmeal-based media supplemented with 5µM Pramipexole. DMSO was used as vehicle. N=30 larvae per treatment/per genotype. Kruskal-Wallis test was used to determine statistical significance. **** - P_{value} < 0.0001.
